# Zearalenone (ZEN) in Livestock and Poultry: Dose, Toxicokinetics, Toxicity and Estrogenicity

**DOI:** 10.3390/toxins12060377

**Published:** 2020-06-07

**Authors:** Jundi Liu, Todd Applegate

**Affiliations:** Department of Poultry Science, University of Georgia, Athens, GA 30602, USA; jundi.liu25@uga.edu

**Keywords:** mycotoxin, zearalenone, modified and masked forms, poultry, swine, ruminants

## Abstract

One of the concerns when using grain ingredients in feed formulation for livestock and poultry diets is mycotoxin contamination. Aflatoxin, fumonisin, ochratoxin, trichothecene (deoxynivalenol, T-2 and HT-2) and zearalenone (ZEN) are mycotoxins that have been frequently reported in animal feed. ZEN, which has raised additional concern due to its estrogenic response in animals, is mainly produced by *Fusarium graminearum* (*F. graminearum*), *F. culmorum*, *F. cerealis*, *F. equiseti*, *F. crookwellense* and *F. semitectums*, and often co-occurs with deoxynivalenol in grains. The commonly elaborated derivatives of ZEN are α-zearalenol, β-zearalenol, zearalanone, α-zearalanol, and β-zearalanol. Other modified and masked forms of ZEN (including the extractable conjugated and non-extractable bound derivatives of ZEN) have also been quantified. In this review, common dose of ZEN in animal feed was summarized. The absorption rate, distribution (“carry-over”), major metabolites, toxicity and estrogenicity of ZEN related to poultry, swine and ruminants are discussed.

## 1. Introduction

Grains are major ingredients for farm animals raised in integration production systems, which primarily contributes energy to the diet. One of the concerns when using grain ingredients in feed formulation is mycotoxin contamination [1]. Previous publications have suggested that about 70% of cereal based feeds are contaminated with at least one mycotoxin [2].

Mycotoxins are secondary metabolites produced by different fungi and are defined as “natural products produced by fungi that evoke a toxic response when introduced in low concentration to higher vertebrates and other animals by a natural route” [3]. The term “mycotoxin” is derived from “mykes” meaning “fungi” and “toxicon” meaning “poison” [4]. Among the approximately 300 to 400 mycotoxins that have been identified, aflatoxin, fumonisin, ochratoxin, trichothecene (deoxynivalenol, T-2 and HT-2) and ZEN are frequently reported mycotoxins due to safety concerns and economic impact [4,5]. ZEN can interact with estrogen receptors in animals and has been defined as an estrogenic mycotoxin and raised additional attention because of its toxicokinetics, toxicity and estrogenicity [6,7,8].

The genus *Fusarium* was established over 200 years ago by Link in 1809 [9]. ZEN or ZEA/ZON (previously known as F-2 toxin), is a non-steroidal estrogenic mycotoxin biosynthesized through a polyketide pathway mainly produced by strains of *Fusarium graminearum (F. graminearum)* (Stob et al. [10] first isolated a uterotrophic compound from corn contaminated with fungus *Gibberella zeae,* also known by the anamorph/asexual name *F. graminearum*), *F. culmorum*, *F. cerealis*, *F. equiseti*, *F. crookwellense* and *F. semitectum* [11,12]. Early researchers partially characterized the toxin ZEN. The report by Urry et al. [13] determined it as an enantiomorph of 6-(10-hydroxy-6-oxo-*trans*-1-undecenyl)-β-resorcyclic acid lactone which they gave the name “Zearalenone”, while the earlier researchers referred to it as F-2 toxin [14,15]. ZEN is a white, crystalline, fat-soluble toxin with a relatively high melting point (164 to 165 °C) [16,17]. It is found in different grains worldwide, including corn, wheat, barley, oats, etc., which are often used as feed ingredients in farm animals [12,18,19].

**Figure 1:**
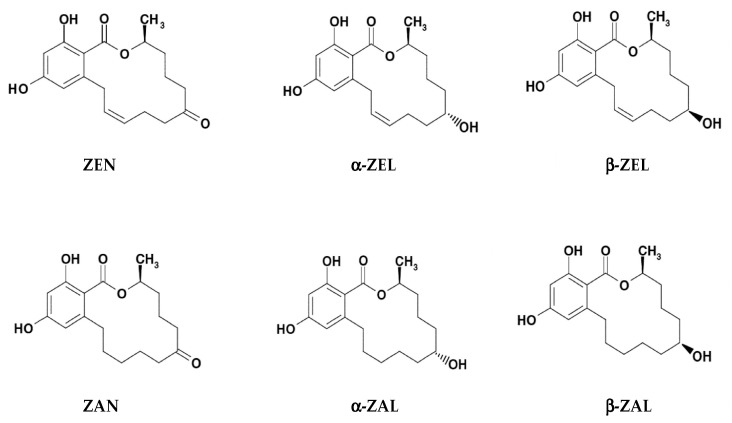
Chemical structures of zearalenone (ZEN/ZEA/ZON) and its modified forms α-zearalenol (α-ZEL/α-ZEN/α-ZOL), β-zearalenol (β-ZEL/β-ZEN/β-ZOL), zearalanone (ZAN), α-zearalanol (α-ZAL), and β-zearalanol (β-ZAL) (modified based upon Urraca et al. [23]).

In both fungi and mammals, the reduction of keto group in ZEN structure leads to two stereoisomeric metabolites α- and β-isomers, while reduction of olefinic double bond leads to the alkane zearalanone [16,20,21]. Common derivatives of ZEN include (Figure 1): α-ZEL (or α-ZEN/α-ZOL), β-ZEL (or β-ZEN/β-ZOL), ZAN, α-ZAL, and β-ZAL [21,22,23]. In feed, ZELs, ZAN and ZALs are the reduced metabolites of ZEN occurring during its phase I metabolism. Other modified and masked forms, including derivatives conjugated with glucose, sulfate and glucuronic acid, occur during its phase II metabolism [16].

Recently, the metabolites and toxicity of modified and masked forms of ZEN have been frequently discussed [16,24,25,26]. Plants can alter the chemical structure of mycotoxins as part of their defense against xenobiotics [24]. However, research data on extractable conjugated and non-extractable bound derivatives of ZEN have not been well established [16,24]. One explanation for the missing adequate number of publications is the undetectable characteristic of these masked derivatives via previous routine analytical procedures [27,28]. The calibrants and reference materials for conjugated forms are still not commercially available [16]. To date, more than 30 modified forms of ZEN have been described, including correspondent *cis*-forms (due to the sunlight exposure) and zearalenone-14-sulfate [16,26,27]. Masked forms derivatives have been often mentioned in literatures including: zearalenone-14-glucoside, α-zearalenol-14-glucoside, and β-zearalenol-14-glucoside [16,24,28,29]. Zearalenone-16-glucoside was another recently reported ZEN glucoside in wheat and barley [30].

ZEN also often co-occurs with other *Fusarium* mycotoxins, mainly deoxynivalenol [31]. After harvest, the production of deoxynivalenol is favored by grain moisture over 20% and temperatures ranges between 21 to 29 °C [32]. Because both ZEN and deoxynivalenol could be produced by *F. graminearum* or *F. culmorum* [12], which means the most suitable moisture and temperature conditions for *Fusaria* growth and production of ZEN are the same that favor deoxynivalenol production [32].

## 2. ZEN Guidance and Concentration in Animal Feed

Mycotoxin contamination in grain ingredients can happen both pre- and post-harvest. The production of mycotoxin by fungi occurs during plant growth, maturity, harvesting, and processing of grains [33]. Multiple factors influence fungal growth and mycotoxin formation, including season, geographical location, drought, harvest time, processing, storage, and distribution [4]. ZEN is mainly formed pre-harvest, however continued fungal growth and ZEN synthesis might continue during poor storage conditions [34].

The first review on the occurrence of mycotoxins dates back to 1977, when it was presented at the first FAO/WHO/UNEP conference on mycotoxins [35]. The contamination of ZEN in grain and animal feed can range from 4 to 11192 µg /kg [36,37]. The regulatory guideline for ZEN varies among different national authorities and regulatory organizations. In this review, the focus remains on the regulatory limits of ZEN in livestock feed (mainly poultry, swine and ruminants). Table 1 shows the regulatory limits for ZEN concentration in complete feed from the European Commission Guidance (EU) and US Food and Drug Administration Guidance [16,31]. Currently, modified and masked forms of ZEN are not considered in the regulations and within the EU guidance [16].

The contamination level of ZEN varies distinctly by the region, country, climate, etc. A recent publication [19] summarized mycotoxin concentrations from more than 74,000 samples from 100 countries over 10 years. Based on the survey results, ZEN is one of the top three mycotoxins in complete animal feed and feedstuffs, with occurrence in 45% of all samples and 55 μg/kg median concentration among the positive samples. ZEN contamination is specific to common feed ingredients used in livestock animal feed ingredients, occurring in corn (44%), corn-DDGS (75%), soybean meal (61%), wheat (33%) and barley (20%). Meanwhile, ZEN was the most prevalent mycotoxin in both soybean and soybean meal samples.

Furthermore, the co-occurrence of mycotoxins in grain samples (containing more than one mycotoxin) has been frequently reported [38,39,40,41]. As ZEN and deoxynivalenol are both produced by *F. graminearum* or *F. culmorum*, the co-occurrence of ZEN and deoxynivalenol was detected in 48% of finished feed, 39% in corn, and 28% in wheat samples (ZEN and deoxynivalenol was also the most frequently observed mycotoxin combination in wheat samples), respectively [19]. This underlines the importance of considering the synergistic effects of multi-mycotoxin contamination. The survey on occurrence of modified forms of ZEN in feed ingredients are less extensive [16]. The co-occurrence of ZEN and ZELs glucosides has been reported, with the sum of modified forms exceeding the ZEN concentration alone by as much as 1.5 times in barley samples and 0.5 times in oat and wheat samples [42].

**Figure 2:**
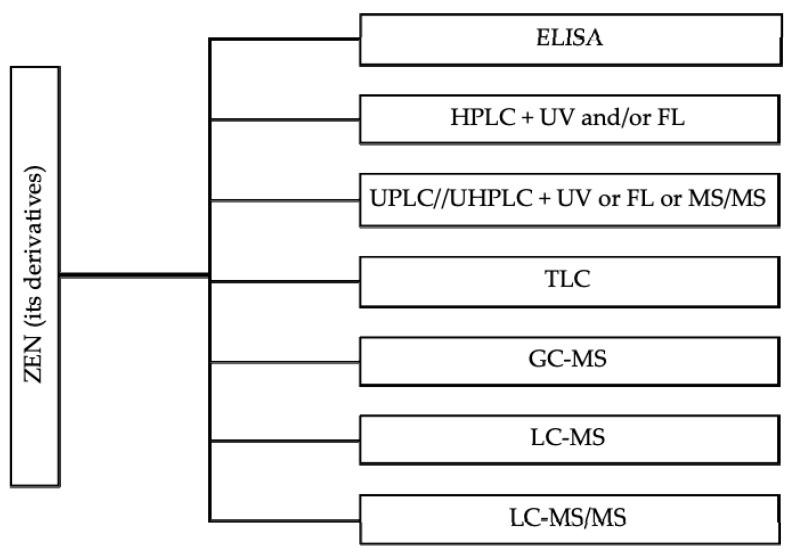
Common analytical methods for the measurement of ZEN and its modified derivatives concentration in animal feed.

Various analytical methods have been well-established to characterize ZEN and its modified forms in feed [43,44,45], including ELISA, LC-MS/MS, HPLC, LC-MS, GC-MS (Figure 2). The common HPLC or UPLC methods are coupled to UV or FL detectors. These HPLC-UV or HPLC-FL methods are based on the use of a combination of acetonitrile, methanol and water as the extraction solvent combined with the used of specific immunoaffinity columns or solid-phase extraction cartridges as clean-up steps [46]. The LC-MS and LC-MS/MS provides information about molecular mass and structural features of components, which are considered more sensitive than other methods in terms of separation, identification and quantification of ZEN [45]. The extraction of modified forms of ZEN is based on the same protocol that have been used to extract ZEN [16,47,48]. Of note, there is a need of calibrants and reference materials for development of validated and sensitive routine methods for modified forms in feed samples, especially with the highly sensitive analytical methods for α-ZEL [16]. A practical option is to conduct quick ELISA test kit for ZEN screening in field situations and to subsequently further validate test results with LC-MS/MS within a laboratory setting.

The occurrence of ZEN has been widely reported in a variety of different countries, regions, years, etc. As mentioned previously, the current review specifically focuses on the concentrations of ZEN and its modified forms in poultry, swine and ruminant feeds. The literature search was conducted using the University Libraries Database at the University of Georgia, PubMed, and Google Scholar search engines with the key words: mycotoxin, zearalenone, feed, occurrence, livestock animal, swine, pig, sow, gilt, poultry, broiler, laying hen, ruminants, dairy, and cattle. Table 2 summarizes the ZEN concentration in selected peer-reviewed surveys, studies and review publications. To date, the research on evaluation of modified and masked forms of ZEN in poultry, swine and ruminant animals are not adequate.

## 3. ZEN Absorption Rate, Distribution (Carry-Over), Metabolites and Excretion in Livestock Animals

ZEN is a heat-stable compound despite its large lactone ring (ZEN in ground corn was stable at 150 °C for 44 h), and degradation was only observed at extremely high temperatures or within an alkaline environment, which makes ZEN thermostable during storage, milling, processing and distribution [16,49,50].

**Figure 3:**
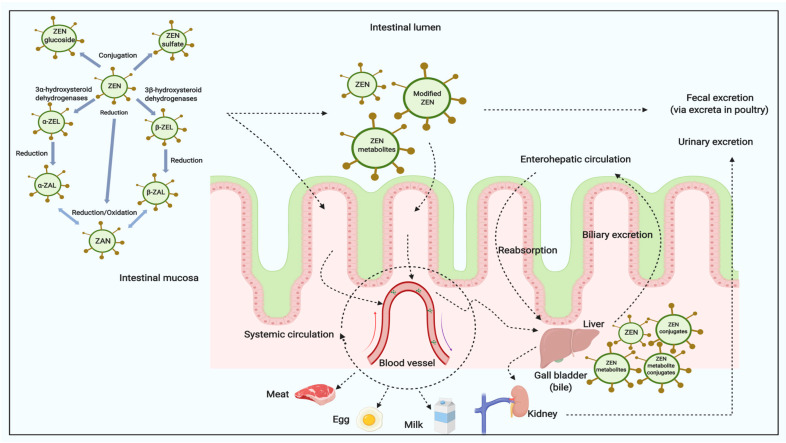
The digestion and metabolism of ZEN and its modified forms in animals (modified based in part upon Dänicke and Winkler [25] and created with Biorender.com).

Once ingested by the animal, ZEN and/or its modified forms are rapidly and extensively absorbed by intestine and modified by liver [12,18,25]. The metabolites (Figure 3) include ZEN, ZELs, ZAN, ZALs and its corresponding conjugates [16,18,23,25,51]. In farm animals, the reductive biotransformation predominates and the resulting metabolites mainly are α-ZAL and β-ZAL, with limited amount of α-ZEL, β-ZEL and other metabolites being produced [16]. The metabolic profile in urine and feces are significantly different among species. For example, higher proportions of an administered ZEN dose were metabolized to α-ZEL in pigs than in cows, whereas ZEN was mainly found as glucuronide conjugates of ZEN and α-ZEL in pig urine [52]. The concentrations of α-ZEL in plasma in some studies may be higher than others; some studies only detected the conjugated form metabolites, while others found both free and conjugated forms of ZEN [18,53,54]. Briefly, among ZEN treated animals, the α-derivatives seem to be the most prevalent in pigs and turkeys, versus β-derivatives which appear to be the abundant metabolites in cattle, goats, broilers and laying hens based on the levels measured in plasma, urine or bile [16]. Very limited amounts of ZEN were detected in liver, kidney, and muscle in animals [25]. In general, there are potentially two major biotransformation pathways for ZEN in animals [54,55]:

(1) hydroxylation to form α-ZEL, β-ZEL, and catalyzed by 3α- and 3β-hydroxysteroid dehydrogenase;

(2) conjugation with glucuronic acid and catalyzed by uridine diphosphate glucuronyl transferase.

Additionally, there is a general consensus that sulfation presents an additional conjugation route for both ZEN and its metabolites [16]. However, limited in vitro data were reported both on the structures and the enzymology of such sulfate derivatives [56]. In the in vivo pig study, zearalenone-14-glucoside, zearalenone-16-glucoside, and zearalenone-14-sulfate were found to be completely hydrolyzed and absorbed in pigs [57].

The high degree of re-absorption in the intestinal tract influences ZEN excretion via enterohepatic circulation [25,54]. Recent publications have relayed investigations on ZEN, α-ZEL and β-ZEL binding to bovine and porcine serum albumins [58,59]. Serum albumin is the most abundant plasma protein in the circulation, which effects tissue distribution and elimination of xenobiotics [58]. The results showed that ZEN can bind to bovine serum albumin with strong intermolecular forces [59]. ZEN binds with higher affinity than α-ZEL and β-ZEL to albumins [58]. ZEN and its metabolites are mostly excreted via the fecal route as glucuronides [16,18].

Previous research demonstrate that ZEN and its reductive metabolites can be detected in both animal tissues and products (Table 3, Table 4 and Table 5). Systemic circulation (Figure 3) of ZEN and its modified metabolites in animals are essentially related to their distributions in different tissues and potential carry-over into animal products [16].

**Table 2:** Summary of ZEN concentration in livestock feed from selected peer-review publications.

Article Type	Feed for Species	ZEN Concentration (µg/kg)	Analysis Method	ZEN Derivatives (α-ZEL, β-ZEL, ZAN, α-ZAL, β-ZAL)	Year
Survey	Poultry; swine	44 to 797; 86 to 629	LC	N.A.	1997 [60]
Study	Poultry	327 to 5850	TLC	N.A.	1998 [61]
Research	Starter pig	200, 400 and 500 (also detected with other mycotoxins)	GC-MS	α, β-ZEL < d.l. (set at 0.2 mg/kg)	2003 [62]
Study	Poultry	0.53	HPLC	N.A.	2004 [63]
Research	Broiler	400, 500, 600 and 700 (also detected with other mycotoxins)	GC-MS	α, β-ZEL < d.l. (set at 0.2 mg/kg)	2004 [64]
Survey	Poultry	3 to 86	HPLC	N.A.	2005 [65]
Research	Weaned pig	300 to 710 (also detected with other mycotoxins)	HPLC	N.A	2005 [66]
Research	Dairy cows	22 and 59 (µg/kg DM)	HPLC	Mean recoveries for α-ZEL and β-ZEL were 81 and 74%; concentration N.A.	2005 [67]
Survey	Laying hen	7.4 to 61.4	HPLC	N.A	2006 [68]
Research	Broiler	70, 3360 and 8280	ELISA	N.A	2008 [69]
Survey	Animal feed	10 to 189	HPLC	N.A	2010 [70]
Research	Post-weaning gilt	100 and 1300 (also detected with other mycotoxins)	ELISA	N.A	2010 [43]
Research	Post-weaning gilt	100 and 1300 (also detected with other mycotoxins)	ELISA	N.A	2010 [44]
Research	Post-weaning gilt	1100, 2000 and 3200	ELISA	N.A	2011 [71]
Survey	Animal feed	Up to 5791	ELISA+HPLC	N.A	2012 [72]
Research	Sow (during gestation and lactation)	Appr. 200, 500 and 1000	Not mentioned	N.A	2012 [73]
Research	Post-weaning piglet	1050 (also detected with another mycotoxin)	ELISA	N.A	2012 [74]
Research	Gilt	200, 400 and 800	GC-MS	N.A	2012 [75]
Survey	Poultry; swine; ruminants	Appr. 12 to 109; 13 to 200; 57 to 194	HPLC and LC-MS/MS	N.A	2014 [76]
Research	Broiler	Appr. 18 and 280	ELISA	N.A	2014 [77]
Research	Dairy cow	24.4 to 112.7 (µg/kg DM; also detected with another mycotoxin)	HPLC	Average recoveries for α-ZEL and β-ZEL were 85% and 86%	2014 [78]
Survey	Broiler	2.22 to 263.51	LC-MS/MS	N.A	2015 [79]
Study	Layer	5.17 to 147.53	HPLC	N.A	2016 [80]
Survey	Swine	36 to 219	HPLC	α-ZEL: <15 to 529; β-ZEL: <11	2016 [81]
Research	Laying hen	400 (also detected with another mycotoxin)	HPLC	N.A	2017 [82]
Case study	Pig (hay pellet)	479	ELISA and LC-MS/MS	α-ZEL: 11.7; β-ZEL: 16.9; ZEN-sulfate: 530; ZEN-glucoside: <d.l.	2018 [83]
Study	Cattle	88.2	UPLC-MS/MS and UPLC-QTOF-MS	N.A	2018 [46]
Study	Duck; pig	39.08 to 47.61; 124.78	HPLC, and LC-MS/MS	For duck feed: α-ZEL:4.19 For pig feed: α-ZAL: 2.31 to 2.48; β-ZAL: 3.11; ZAN: 4.17 to 6.69	2018 [45]
Research	Pig	Appr. 800	UPLC	N.A	2018 [84]
Research	Turkey	470 (also detected with other mycotoxins)	HPLC-MS/MS	N.A	2019 [85]
Research	Broiler	Appr. 2000 (also detected with other mycotoxins)	ELISA	N.A	2019 [86]
Research	Broiler	280 to 520	SIDA-UHPLC-MS/MS	N.A	2020 [87]

### 3.1. Poultry

For poultry, researchers have found both ZEN and reductive ZELs metabolites (α-ZEL and β-ZEL) can be detected in blood, liver, kidney, muscle, intestine and excreta [88,89]: ZEN administered via both intravenous injection and oral administration at a dose of 1.2 mg/kg b.w. was measurable from 5 min to 2 h in plasma and was rapidly transformed into α-ZEL and β-ZEL in plasma of broilers. Other research [90] also revealed the metabolites of ZEN in blood in different poultry species, with a higher production of β-ZEL than the α-ZEL in broilers and layers, versus turkey poults, which were more efficient at bio-transformation of ZEN to α-ZEL. ZEN was measurable up to 1 h in the liver, kidney, and small intestine. α-ZEL and β-ZEL were detectable up to 12 h in the liver, kidney and small intestine, whereas both were only detectable up to 1 h in muscle following oral administration. The concentration of α-ZEL and β-ZEL in different tissues is as follows: small intestine > liver > kidney > muscle. In poultry, bile has been reported to play an important role as an excretory route for ZEN and its metabolites [91]. Besides, ZEN, α-ZEL and β-ZEL were detected in excreta up to 12 h after oral administration, with the concentration α-ZEL > β-ZEL [89]. Another study fed birds diets contaminated with 0.4 mg/kg ZEN feed from d 29 to 84 and did not find ZEN carry-over into the liver, whereas α-ZEL was detected on the last sampling day [87]. The carry-over of α-ZEL may be possible due to the fact that ZEN is mostly and rapidly eliminated in excreta [88]. This was supported by the excreta results: when they measured the excreta ZEN concentration collected within the last two days, the ZEN concentration was 0.27 mg/kg. In a laying hen study [92], researchers found that α-ZEL was detectable in the liver, whereas ZEN was not detected in either breast meat or the liver. Dailey et el. [93] found after a single dose exposure of ZEN (10 mg/kg) in laying hens, it is possible that significant levels of ZEN lipophilic metabolites might accumulate in egg yolk if the exposure time was prolonged. Another study was conducted to assess the carry-over of ZEN into eggs [92], and researchers reported that no detectable ZEN was detected in eggs from commercial production [12]. The metabolites and carry-over effects of dietary ZEN in poultry from previous publications are shown in Table 3.

### 3.2. Swine

Biehl et al. [54] reported that the absorption rate of ZEN in pigs was estimated to be 80–85% following a single oral dose of 10 mg ZEN/kg b.w. The estimated biological half-life of radiolabeled ZEN was 87 h in the intact pig, whereas it was reduced to 3.3 h when the bile of the pig was removed. Additionally, 45% of the administered dose was recovered in urine and 22% in feces within the first 48 h. After absorption, ZEN and its metabolites could be detected in the liver, bile, plasma, urine, digesta and feces [94]. Earlier study showed that ZEN and its metabolites can be detected in plasma around 30 min after oral administration [54,55]. In swine, the major metabolites are glucuronide conjugates of ZEN and α-ZEL [54]. Gajęcka et al. [95] surmised that α-ZEL is the predominant metabolite of ZEN in pigs and the low dose of ZEN could alleviate inflammation in the digestive tract (especially in the proximal and distal intestinal tract), and could increase body weight gains in gilts. The ZEN was reduced to α-ZEL and β-ZEL when incubated with homogenized intestinal mucosa from sows in the presence of nicotinamide adenine dinucleotide phosphate. Additionally, the rate of glucuronic acid conjugation of ZEN was about 30-fold higher than that of reduction in the presence of UDP glucuronic acid [96]. The β-ZEL was detected only in bile of gravid sows, and when fed a high (0.42 mg/kg) concentration of ZEN in female pigs [97,98]. In addition, researchers have reported that the main route for excretion of ZEN and its metabolites in swine is through urine, which was twice as high as the amount eliminated through feces [54,57]. Research found that in gilts the cumulative recovery of ZEN and α-ZEL in duodenal digesta and urine was 35% and 70% after 72 h, respectively (as a percentage of total ZEN administrated). Additionally, 14 days after the bolus injection, both ZEN and α-ZEL were lower than the detection limit in the bile, liver and urine. The elimination half-time of ZEN in excreta was 2.63 h. This is to say that ZEN is completely eliminated from gilts within this period of time with a massive single bolus [94]. The metabolites and carry-over effects of dietary ZEN in swine from previous publications are shown in Table 4. ZEN is mainly converted to α-ZEL in pigs, and exposure risk to humans by consumption of edible product is negligible compared to direct consumption of grain-based food [99].

### 3.3. Ruminants

For ruminants, prior reports noted that β-ZEL was the predominant ZEN metabolite in urine, and the free and conjugated ZEN can both be detected in cows’ milk [52]. In a trial with one single cow, researchers found that 0.7% of ZEN could carry-over into the milk when feeding up to 200 mg ZEN/day in the feed for 7 days [100]. This carry-over into milk normally occurs when animal ingests a high dose of ZEN in feed. Other researchers [78] have also demonstrated that ZEN is mainly metabolized to β-ZEL, and less extensively to α-ZEL in bovine species. The authors could detect the ZEN, α-ZEL and β-ZEL in bile. The concentrations of ZEN, α-ZEL, β-ZEL, ZAN, α-ZAL and β-ZAL in serum, urine and milk were lower than 1, 1, 4, 100, 50 and 200 ng/g, respectively. The concern for ZEN carry-over in ruminants may be minor based on their endogenous ruminal detoxification [101]. In addition, the health status and blood-milk barrier would also affect the transfer of ZEN into milk [102]. Seeling et al. [67] conducted a trial and found that different feed intakes could affect the sum of all ZEN metabolites and the proportion of β-ZEL in dairy cows. In a sheep study with two adult ewes (30-35 kg), researchers noted that sheep were capable of metabolizing ZEN with further reduction of the C11-C12 double bond, which led to the α-ZEL and β-ZEL [103]. In another study with goats [104], blood plasma, urine, and feces samples were collected consecutively after intravenous injection of ZEN at doses of 2.4 mg/kg and 1.2 mg/kg b.w. The distribution half-life and elimination half-life of ZEN were 3.15 and 28.58 h, respectively. ZEN, α-ZEL, and β-ZEL were detected in both urine and feces, with β-ZEL being the predominant metabolite. Additionally, ZEN and its metabolites were largely in their glucuronide and/or sulphate conjugated forms in urine, while they were largely in free forms in feces. The metabolites and carry-over effects of dietary ZEN in ruminant animals from previous publications are shown in Table 5. ZEN and its metabolites can be detectable in liver and bile, but in most studies are not detected in the milk [67,78].

**Table 3:** Metabolites and carry-over of dietary ZEN in Poultry.

Species	ZEN Concentration (mg/kg of Diet, Fed-Basis)	Duration (Days)	Metabolites and Carry-Over into Tissues (ug/kg or ug/L)	Remarks	References
Laying hen	^14^C-ZEN: 10 mg/kg b.w.	Single bolus	Leg, wing, breast muscle: very low radioactivity; Yolk: appr. 2000 ug/kg after 72 h	94% of ^14^C radioactivity eliminated via excreta within 72 h of dosing; ZEN was readily conjugated with glucuronic acid	[93]
Broiler	^3^H-ZEN: 5 mg/kg b.w. (appr. 50 mg/kg diet)	Single bolus	Muscle: relatively low, ZEN max. 111 at 24 h after dosing (α-ZEL, β-ZEL n.d.); Liver: total ZEN α-ZEL and β-ZEL 17-2543 within 24 h; Rapid clearance	Conjugated n.d.	[88]
Turkey	800	14	Liver: ZEN 282; α-ZEL 2720; Kidney: ZEN 120, α-ZEL 480; β-ZEL traces in liver and kidney (after incubation with β-glucuronidase and sulfatase)	ZEN and α-ZEL mainly conjugated in plasma and conjugates consisted of both glucuronides and sulfate conjugates	[105]
Chicken	10 mg/kg b.w.	20	Liver: ZEN 207; Kidney: ZEN 416; Muscle: ZEN 170	Metabolites and conjugated n.d.	[106]
Laying hen	1.58	112	Liver: α-ZEL 3.5-3.8 (36% free, 28% conjugated with glucuronic acid, and 36% with sulphate); ZEN<1-3.2 (46% free, 54% conjugated with glucuronic acid, and <5% with sulfate); n.d. residues in egg yolk, albumen, breast muscle, abdominal fat, ovary and follicles, magnum		[92]
Pekin duck	Up to 0.06	49	Liver: ZEN, α-ZEL and β-ZEL<d.l. kidney (after incubation with β-glucuronidase and sulfatase)	In bile, dose-response related increase in ZEN, α-ZEL and β-ZEL-concentrations; the mean proportions of ZEN, α-ZEL and β-ZEL of the sum of all three metabolites were 80, 16 and 4% respectively	[91]
Turkey	Up to appr. 0.04	35	Plasma, liver or breast meat: ZEN or its metabolites n.d.	In bile, concentrations of ZEN and α-ZEL increased with dietary ZEN concentration; the mean proportions of ZEN, α-ZEL and β-ZEL of the sum of all three metabolites were 19, 77 and 4% respectively	[107]
Broiler	0.3 mg/kg b.w.	Single bolus (intravenously and orally)	Plasma: ZEN and its metabolites n.d.		[108]
Broiler (Female)	1.2 mg/kg b.w.	Single bolus (orally)	Liver: ZEN 3.52; α-ZEL 7.84-105.2, β-ZEL 24.4-30.9; Kidney: ZEN 3.55; α-ZEL 1.63-77.99, β-ZEL 4.8-36.6; Muscle: α-ZEL 2.55, β-ZEL 2.40; (after incubation with glucuronidase/arylsulfatase)	Time-response decrease in ZEN, α-ZEL and β-ZEL concentration	[89]
Broilers (male; slow-growing breed)	0.4	56	Liver: ZEN n.d.; α-ZAL n.d.; α-ZEL 0.4-0.8 (5 out of 8 samples)		[87]

**Table 4:** Metabolites and carry-over of dietary ZEN in Swine.

Species	ZEN Concentration (mg/kg of Diet, Fed-Basis)	Duration (Days)	Metabolites and Carry-Over into Tissues (ug/kg or ug/L)	Remarks	References
Pig (female, 8-11 kg b.w.)	40	28	Liver: ZEN 128; α-ZEL 94 and β-ZEL <d.l.	Conjugates n.d.	[109]
Piglet (appr. 18kg b.w.)	0.5 mg/kg b.w.	Single bolus	Liver, kidney, muscle: ZEN, α-ZEL and β-ZEL<d.l. (after incubation with glucuronidase)	ZEN: d.l., α-ZEL and β-ZEL: 0.8-9.2 ug/kg	[110]
Pig (appr. 50 kg b.w.)	1)ZEN: 0.25 2)ZEN: 0.25+OTA 0.1	90	1) liver, kidney, muscle, adipose tissue: ZEN and α-ZEL < d.l. 2) liver, kidney: α-ZEL-traces (max. 4 ug/kg after incubation with glucuronidase), ZEN<d.l.; muscle and adipose tissue: ZEN and ZAN<d.l.		[111]
Pig (appr. 70 kg b.w.)	0.7	18	Liver: ZEN<d.l. -3.1; α-ZEL 3.6-12; β-ZEL 1.9 -4.8; Muscle: α-ZAL up to 13.3; α-ZEL up to 14.5; traces of ZEN and β-ZAL; ZEN and ZAN <d.l.		[112]
Piglet (appr. 33 kg b.w.)	0.01; 0.06; 0.15; 0.22; 0.42	35	Liver (after incubation with β-glucuronidase and sulfatase) 1.8 ZEN + 0.3 α-ZEL; 0.2 ZEN + 0.1 α-ZEL; 2.1 ZEN + 1.1 α-ZEL; 2.9 ZEN + 1.7 α-ZEL; 5.3 ZEN + 2.8 α-ZEL		[113]
Piglet (appr. 33 kg b.w.)	1 mg/kg b.w.	Single bolus	Liver (14 days after the bolus, after incubation with β-glucuronidase and sulfatase): ZEN, α-ZEL and β-ZEL<d.l.		[94]
Pig (female and barrows)	0.056	84	Liver: only α-ZEL was detected with mean carry-over factors (averaged over all group) of 0.0094; ZEN, α-ZEL and β-ZEL n.d. in serum	Residues of ZEN + α-ZEL + β-ZEL was positively correlated in liver and bile (Liver showed 0.9% carry-over ratio)	[99]

**Table 5:** Metabolites and carry-over of dietary ZEN in Ruminants.

Species	ZEN Concentration (mg/kg of Diet)	Duration (Days)	Metabolites and Carry-Over into Tissues (ug/kg or ug/L)	Remarks	References
Lactating cow	0.39-1.93 mg/kg concentrate	49	Muscle, liver, kidney, milk: ZEN<4		[114]
Lactating cow	5000 mg/animal	Single bolus	Milk: ZEN and β-ZEL: traces (<1)	incubation with β-glucuronidase	[115]
Lactating cow	1800 mg/animal	Single bolus	Milk: ZEN and β-ZEL: 1-2	incubation with β-glucuronidase	[115]
Lactating cow	25	7	Milk: 1360 ug/l total residues of ZEN, α-ZEL, β-ZEL, free and conjugated	0.7% of consumed ZEN recovered with milk	[52]
Lactating cow	50-165 mg/day; 545 mg/day; 1800 or 6000 mg/animal	21; 21 and single bolus	Milk: ZEN, α-ZEL and β-ZEL and conjugates < d.l.; Milk: ZEN max. 2.5; α-ZEL max. 3.0 (only as conjugates, incubation with β-glucuronidase/aryl sulfatase); Milk: ZEN max. 4.0 or 6.1; α-ZEL max. 1.5 or 4.0; β-ZEL max 4.1 or 6.64		[116]
Lactating cow	25 or 100 mg/day	6	Milk: ZEN-equivalents max. 0.4 or 1.2 (by ELISA after incubation with β-glucuronidase)		[117]
Lactating cow	0.02-0.05 mg/kg dry matter	63	Milk: ZEN and α-ZEL<0.5 (after incubation with β-glucuronidase)		[118]
Dairy cow	0.05 mg/kg dry matter	28	Milk: ZEN, α-ZEL, β-ZEL, ZAN, α-ZAL, β-ZAL < d.l.		[67]
Goat	2.4 and 1.2 mg/kg b.w.	Single bolus (intravenously)	Liver: α-ZEL 5.2 and β-ZEL 4.5 at 48 h poste administration	The proportion of conjugated α-ZEL and β-ZEL were appr. 29 and 41% respectively	[104]
Dairy cow	0.02 to 0.11 mg/kg dry matter	28 wks	Milk: ZEN, α-ZEL, β-ZEL, ZAN, α-ZAL, β-ZAL < d.l	Bile: ZEN, α-ZEL, and β-ZEL were detectable (bile can be regarded as an indicator for dietary ZEN-exposure)	[78]

## 4. ZEN Toxicity and Estrogenic Effect in Livestock Animals

### 4.1. Toxicity

Generally, ZEN has low acute toxicity to animals at low concentrations. However, previous publications elaborated the sub-acute, sub-chronic, chronic immunotoxicity, genotoxicity, productive and developmental toxicity, as well as endocrine disturbance effects caused by ZEN [12,51,59]. The fate and adverse effects of ZEN are partly determined by the processes of elimination, which is closely related to the biliary excretion and enterohepatic cycling [54,119]. Based on JECFA, the safety of ZEN can be evaluated based on the dose that had no hormonal effects in pigs, which is known as the most sensitive animal species to ZEN [120].

Poultry seem to be quite tolerant to ZEN, which may be explained by the naturally high concentration of estrogen in poultry blood. Natural estrogens are considered to have higher receptor affinity compared to the *Fusarium* toxins [101]. ZEN had no effect on feed intake or body weight gain on young male turkey poults. In contrast, feeding ZEN-contaminated diets to turkey poults lead to strutting behavior, increased size and coloration of caruncles and dewlaps, and swollen vent tissue [105]. An additional study indicated that purified ZEN may increase oviduct development in growing female chickens and delay growth of the testes in young male chickens [121].

Feeding female pigs with 1.3 mg/kg ZEN diet can reduce platelets, haemoglobin, globulin, triglycerides and high-density lipoproteins in serum; increase enzyme activities; and lead to degeneration of the liver and kidney [43]. In addition, the difference in susceptibility to the estrogenic effects of ZEN was also not related to the circulation difference of ZEN and its metabolites [1,54].

For ruminants, ZEN may lead to lower conception rates in heifers [122]. However, the contribution of ZEN to the susceptibility difference is unknown, because most of the related research has been conducted in pigs [89]. One possible explanation for the differences related to species’ susceptibility may be related to the variation in the number and affinity of estrogen receptors. In general, pigs and sheep are more susceptible species than poultry [16].

Last but not least, the toxicity of modified and masked forms of ZEN (both extractable conjugated and non-extractable bound forms) has not been adequately evaluated [16,26,123]. These modified forms of ZEN are not detected by routine analytical procedures [16,27,28]. Toxicological data related to these undetectable forms of ZEN are scarce, which implies that analysis of samples containing these compounds leads to an underestimation [24]. Study has found zearalenone-glucoside can be hydrolyzed during digestion in 27 kg female pigs [28]. A more recent study has found that the estimated oral bioavailability of ZEN was 61 to 85%. The α-ZEL and β-ZEL were completely absorbed after the oral administration. The absorbed fraction of zearalenone-14-glucoside was estimated to be 61%, which suggested complete hydrolysis and absorption of zearalenone-14-glucoside. The authors indicated that α-ZEL, β-ZEL, zearalenone-14-glucoside, zearalenone-14-sulfate contribute to the total systemic toxicity of ZEN in pigs and should be taken into consideration for the risk assessment [124]. It is important to account for the total concentration of ZEN and its modified forms [16].

### 4.2. Estrogenicity

The estrogenic factor has been recognized as early as late 1920s [125]. Since then, the association between the consumption of moldy grains and hyper-estrogenism in pigs has been frequently observed and reported [126,127,128]. ZEN is constituted from phenol derivatives and passively crosses the cell membrane [16]. The special hormonal-like response can mimic the endogenous steroidal sex hormone 17-β-estradiol actions after binding to estrogen receptors and effect the estrogen signaling pathway in animals [129]. These responses could result in permanent pathologic alterations of the reproductive tract, which can cause infertility at high intake levels, particularly in prepuberal gilts, leading to pseudopregnancy, infertility, increased embryo lethal resorptions, swollen edematous vulva, vaginal/rectal prolapse, and reduced litter size (due to fetal resorption and implantation failure) [1,12,16,54,55,107,113,130]. The α-ZEL and β-ZEL are two common metabolites of ZEN that relate to the hormonal and estrogenic effects. The estrogenic activity of α-ZEL was 3 to 100 times higher than ZEN [89]. Le Guevel and Pakdel [131] found that α-ZEL was 17 times stronger versus α-ethinyl estradiol with three different bioassays using estrogen receptor gene activation. ZEN binds to estrogenic receptors and has a stronger affinity to α- than to β-estrogenic receptors [16]. Based on the “uterotrophic activity” assed in rodents, the estrogenic activity of ZEN and its modified forms are classified in order: α-ZEL > α-ZAL > ZEN ≈ ZAN ≈ β-ZAL > β-ZEL [16]. Other researchers claimed that the major metabolites known to have affinities for estrogenic receptors are in the following order: α-ZAL > α-ZEL > β-ZAL > ZEN > β-ZEL [87]. The risk of hyper-estrogenic effects for α-ZEL is underestimated, because it is neither often determined or regulated [24]. Little is known about the metabolic fate of α-ZAL, which is used legally as a growth promoter in some countries with the name zeranol [16]. Poultry only respond to the presence of ZEN at extremely high concentrations. Cattle are more resistant to the estrogenic effect of ZEN because they bio-transform ZEN more into β-ZEL than α-ZEL [16].

Recently, the estrogenic activity of metabolites hydrolyzed zearalenone and decarboxylated hydrolyzed zearalenone formed by hydrolysis of ZEN has raised attention as a potential ZEN degradation strategy in animal feed [132]. Additionally, ZEN and its reduced forms are competitive substrates for 3α-hydroxysteroid dehydrogenase and 3β-hydroxysteroid dehydrogenase enzymes, which effect the synthesis of steroids [133].

Concentrations of 1-5 mg/kg of dietary ZEN have been reported to be sufficient to cause clinical signs [134] and hyper-estrogenic clinical signs at 1 mg/kg in pigs [135]. In a preliminary trial [53], researchers reported that prepubertal female pigs fed 0.25 mg/kg of ZEN resulted in redness and swelling of the vulva, swelling of the mammaries and numerous vesicular follicles and some cystic follicles on the ovaries, versus pigs fed 0.05 mg/kg ZEN. However, after ingestion of diets with 0.05 or 0.25 mg/kg ZEN, both treatments showed higher numbers of vesicular follicles on the ovaries when compared to pigs fed the control diets without the mycotoxin. Gilts fed with 1.1 mg/kg ZEN diet showed increased vulva length, vulva width, vulva height and vulva area compared with gilts fed a control diet [71]. Similar results were found by feeding gilts with 0.2, 0.4, or 0.8 mg/kg ZEN contaminated diets and noted that dietary ZEN linearly increased vulva size (width, length and area) [75]. Patience et al. [32] demonstrated that feeding gilts with 1-3 mg/kg ZEN for 3 to 7 days, can lead to hyper-estrogenism and prolapses in prepubertal gilts; with 3 to 10 mg/kg ZEN in the middle of the estrus cycle leading to anestrus and pseudopregnancy; with 15 to 30 mg/kg during the first trimester of pregnancy leading to early embryonic death and reduced litter size. Additionally, the prevalence of ZEN may also cause infertility in boars, with atrophied testes and enlarged mammary glands [32,130]. These symptoms can normally be alleviated after the replacement with clean feed in practice.

## 5. Conclusions

The concern of mycotoxin ZEN in relation to livestock animals is vital:

(1) The occurrence of ZEN and co-occurrence of ZEN with other mycotoxins in grain and complete feed is still relatively high;
(2) The amount of ZEN that carries over into final animal products (meat, egg, milk) is very limited under normal farming systems. However, ZEN and its modified metabolites can be detected in blood, liver, gut, urine and feces;
(3) The special structure of ZEN mimics the effects of estrogen, which closely relates to the reproductive functionality of livestock, with swine being the most sensitive species.
(4) Modified and masked forms of ZEN should be included and taken into consideration for the risk assessment of ZEN for farm animals.

In summation, sub-clinical doses of ZEN may not significantly influence on the productive performance of livestock and poultry, however, the continuous consumption of ZEN contaminated feed could lead to a detectable amount of ZEN and/or its metabolites in the blood, liver, intestine content, urine and feces.

## Figures and Tables

**Table 1:** European Commission Guidance (EU) and US Food and Drug Administration Guidance (FDA) guidance values for ZEN concentration in complete feed [16,31].

Item	Species		ZEN (µg/kg)
EU	Poultry	-	-
	Swine	Sows and fattening pigs	250
		Piglets and gilts	100
	Ruminants	-	500
FDA	Poultry	-	No guidance levels
	Swine	Sows and fattening pigs	
		Piglets and gilts	
	Ruminants	-	

## Abbreviations

ZEN, zearalenone; α-ZEL, α-zearalenol; β-ZEL, β-zearalenol; ZAN, zearalanone; α-ZAL, α-zearalanol; β-ZAL, β-zearalanol; OTA, ochratoxin A; ELISA, enzyme-linked immunosorbent assay; HPLC, high-performance liquid chromatography; UV, ultraviolet; FL, fluorescence; LC, liquid chromatography; MS, mass spectrometer; UPLC, ultra-performance liquid chromatography; TLC, thin layer chromatography; QTOF, quadrupole-time-of-flight; b.w., body weight; appr., approximate; n.d., not determined; d.l., detection limit.
